# A first study comparing preservation of a ready‐to‐eat soup under pressure (hyperbaric storage) at 25°C and 30°C with refrigeration

**DOI:** 10.1002/fsn3.212

**Published:** 2015-02-26

**Authors:** Sílvia A. Moreira, Pedro A. R. Fernandes, Ricardo Duarte, Diana I. Santos, Liliana G. Fidalgo, Mauro D. Santos, Rui P. Queirós, Ivonne Delgadillo, Jorge A. Saraiva

**Affiliations:** ^1^ QOPNADepartamento de QuímicaUniversidade de AveiroCampus Universitário de Santiago3810‐193AveiroPortugal

**Keywords:** Food preservation, high pressure, hyperbaric storage, microbial loads, refrigeration, soup

## Abstract

Hyperbaric storage (HS), storage under pressure at 25°C and 30°C, of a ready‐to‐eat (RTE) soup was studied and compared with refrigeration. Soup was stored at different time (4 and 8 h), temperature (4°C, 25°C, and 30°C), and pressure (0.1, 100, and 150 MPa) conditions, to compare microbial loads and physicochemical parameters. HS resulted in similar (microbial growth inhibition) to better (microbial inactivation) results compared to refrigeration, leading to equal and lower microbial loads, respectively, at the end of storage. Lower/higher pressure (100 vs. 150 MPa) and shorter/longer storage times (4 vs. 8 h) resulted in more pronounced microbial growth inhibition/microbial inactivation. Aerobic mesophiles showed less susceptibility to HS, compared to *Enterobacteriaceae* and yeast and molds. HS maintained generally the physicochemical parameters at values similar to refrigeration. Thus, HS with no need for temperature control throughout storage and so basically energetically costless, is a potential alternative to refrigeration.

## Introduction

Since 1899, with the first study about high pressure processing (HPP) application in food (Hite 1899), this technology is being increasingly applied for nonthermal pasteurization and shelf‐life extension of a variety of commercialized food products (Norton and Sun 2008). For this purpose, pressure ranges between 400 and 600 MPa, short periods (a few seconds up to 20 min), and mild temperatures are used (Butz and Tauscher 2002; Ramirez et al. 2009). These pressures have the ability to inactivate microorganisms with minimal effects on food quality.

In addition to nonthermal pasteurization, in the last years high pressure has also raised interest for several other applications, as for example, to modify the properties of macromolecules like cellulose (Figueiredo et al. 2010), food proteins (Correia et al. 2011), and modulate physiological processes (Saraiva and Rodrigues 2011). Another possible application, very recently reported, is food preservation under pressure at and above room temperature conditions, as a possible basically energetically costless alternative to refrigeration.

The first evidence of the viability of preserving foods under pressure for long time periods (hyperbaric storage) appeared by chance, with the recovery of the submarine Alvin, where some consumable food products (bouillon, apples, and sandwiches) were found after 10 months at 1540 m depth (≈15 MPa) and 4°C (Jannasch et al. 1971). Further, a study on codfish showed that when stored under pressure (24 MPa at 1°C) for 21 days, the fish maintained the fresh quality, without microbial growth, whereas samples stored at 0.1 MPa were classified as unacceptable (Charm et al. 1977). Nevertheless, these two cases still require energy to maintain the refrigerated temperature during all the storage period.

Recently, it was proposed a new food preservation methodology, hyperbaric storage (HS) at room temperature (Segovia‐Bravo et al. 2012; Fidalgo et al. 2014; Queiros et al. 2014; Santos et al. 2014). This method has advantages relatively to refrigeration, since there is no need for temperature control and energy is only needed during compression (until reaching the required pressure level) and decompression, allowing to maintain food products' quality under pressure with great energy savings.

The first study was reported by Segovia‐Bravo et al. (2012), concerning HS (under 25, 100, and 220 MPa at 20°C, for 15 days) of strawberry juice, showing the possibility to extend this product's shelf life, without quality changes and with microbial growth inhibition and inactivation, when compared to the juice stored at atmospheric pressure at the same temperature and under refrigeration. However, this study was performed with an acid food, with acidity contributing for a certain degree of microbial stability of this food. As far as we are aware, only two other publications are available in the literature, using highly perishable food products (high pH and water activity), watermelon and melon juices (Fidalgo et al. 2014; Queiros et al. 2014). Watermelon juice stored under 100 MPa, with no temperature control, at naturally variable room temperature conditions (18–21°C) showed not only an inhibition effect on microbial growth but also a reduction in the microbial load, when compared to the initial value and refrigeration (Fidalgo et al. 2014). Moreover, a similar behavior was observed in melon juice for temperatures at and above room temperature. This product was stored under 25–150 MPa at and above room temperatures (25°C, 30°C, and 37°C) for 8 h, having been observed a microbial growth inhibition (only above 25 MPa), while pressures of 100–150 MPa caused a reduction in the initial microbial loads, to values below those verified for refrigeration storage (Queiros et al. 2014).

This hyperbaric storage principle is very interesting, since it opens the possibility to preserve food products with no temperature control and so with no energetic costs that are necessary for refrigeration. As a novel conceptual possible food preservation methodology, it needs to be further studied for more food products, to gain broader and deeper knowledge about its full potential application.

Soup is considered a universal dish with different textures, tastes, and forms and has a usual pH close to neutral (5–6) and high water activity (>0.90), making this product highly perishable, needing refrigeration for its preservation (Pinilla et al. 2005; Shibeshi and Farid 2011) and was chosen as a case study of a ready‐to‐eat (RTE) food for the present work.

The aim of this work was to study the feasibility of soup storage under pressure, at room‐like temperatures (25°C and 30°C), as a case study of a highly perishable RTE food product. Two pressure levels (100 and 150 MPa) combined with two different storage periods (4 and 8 h) and temperatures (25°C and 30°C) were studied. Microbial load (total aerobic mesophiles, *Enterobacteriaceae*, and yeast and molds) and physicochemical parameters (pH, titratable acidity, reducing sugars, and color) were analyzed. The results were compared with soup stored for the same time period at the same temperatures at atmospheric pressure (0.1 MPa), and under refrigeration (4°C).

## Materials and Methods

### Chemicals

The chemical 3,5‐dinitrosalicylic (DNS) acid was acquired from Acros (NJ, USA). Plate count agar (PCA), Violet Red Bile Dextrose agar (VRBDA), and Rose Bengal Chloramphenicol agar (RBCA) were purchased from Merk (Darmstadt, Germany).

### Preparation of soup samples

Fresh refrigerated precooked carrot soup (prepared with water, potato, carrot (25%), onion, turnip, leek, garlic, salt, and olive oil) was purchased from a local supermarket. The soup was divided into different aliquots (10 g) and immediately frozen and stored at −80°C until use to minimize possible difference between samples. Before each experiment samples were thawed at 4°C.

### Storage experiments

Hyperbaric storage experiments were carried out on a hydrostatic press (High pressure system U33; Institute of High Pressure Physics, Warsaw, Poland). This equipment has a pressure vessel of 35 mm inner diameter and 100 mm height surrounded by an external jacket, connected to a thermostatic bath (Huber Compatible Control CC1, Huber, NJ, USA) to control the temperature. A mixture of propylene glycol and water (40:60) was used as a pressurizing fluid and to control the temperature in the external jacket. The soup samples were placed in low permeability polyamide–polyethylene bags (PA/PE‐90; Albipack – Packaging Solutions, Águeda, Portugal) and the bags were heat‐sealed manually with care to avoid as much as possible to leave air inside the bags. Each bag containing the soup was afterward inserted in a second bag (using the same type of low permeability polyamide–polyethylene bags) that was heat‐sealed under vacuum. Different preservation combinations were performed for two different periods (4 and 8 h) under two pressure levels (100 and 150 MPa) at 25°C and 30°C. Control samples were maintained at atmospheric pressure (0.1 MPa): (1) one control was stored during the same time and under the same temperature conditions; (2) and the second control was maintained during the same time and under refrigeration (4°C). Both controls, described above, were immersed in the same fluid of the pressurized samples, and kept in the dark to mimic the conditions of the samples under pressure, except for pressure.

### Microbial analyses

After each period of storage, 2.0 g of aliquots were obtained aseptically and homogenized with 18.0 mL of Ringer's solution. From the 10^−1^ dilution, other decimal dilutions were prepared (up to a dilution of 10^−4^ allowing a maximum microbiological quantification of 6.0 Log_10_ CFU/g). Soup naturally occuring flora was analyzed by: Total aerobic mesophiles (TAM) counts quantification in PCA after incubation at 30 ± 1°C for 72 ± 3 h; *Enterobacteriaceae* (ENT) counts quantification in VRBDA, being incubated at 37 ± 1°C for 24 h; and Yeast and molds (YM) quantification on RBCA after incubation at 25 ± 1°C for 5 days. Petri dishes with 15–300 colony‐forming units (CFU) were considered for quantification and the results were expressed as logarithmic of CFU per gram of soup (Log_10_ CFU/g).

### Physicochemical analyses

#### pH and titratable acidity

Soup (2.0 g) was mixed with 8.0 mL of distilled water with an Ultraturrax T25 homogenizer (Janke & Kunkel IKA‐Labortechnik, Staufen, Germany). The pH value of the samples was measured at 20°C with a properly calibrated glass electrode (Crison, Barcelona, Spain).

The solution was titrated with 0.02 mol/L sodium hydroxide solution to pH 8.1, using an automatic titrator (Crison Titromatic 1S; Crison, Barcelona, Spain) which was calibrated with pH 4.0 and 7.0 buffers. Titratable acidity was expressed in mg malic acid/100 g soup.

#### Reducing sugars

One gram aliquot of soup was diluted in 20 mL of distilled water at room temperature and homogenized for 15 sec with an Ultraturrax T25 homogeniser. The mixture was centrifuged and to test tubes were added 1.0 mL of supernatant soup and 1.0 mL of DNS reagent. The mixture was heated to 100°C for 5 min followed by cooling. Absorbance at 540 nm was measured using a Multiskan Go microplate spectrophotometer (Thermo Scientific) with Brand plate of 96 wells. The reducing sugars' values were expressed in mg of glucose equivalents/g of soup, using a calibration curve previously built.

#### Color

The color parameters a* (red/green color), b* (yellow/blue color), and L* (lightness) were determined using the CIE*Lab* space, at 25°C. The absorption spectra were recorded using a spectrophotometer Konica Minolta CM 2300d (Minolta Konica, Tokyo, Japan). The CIE*Lab* parameters were determined using the original SpectraMagic^™^ NX Software, Konica Minolta, according to regulations by the International Commission on Illumination. The total color difference, ΔE* was calculated by Eq. (1):

(1) $$\Delta E^{*}={\left[{\left(L^{*}-L_{0}^{*}\right)}^{2}+{\left(a^{*}-a_{0}^{*}\right)}^{2}+{\left(b^{*}-b_{0}^{*}\right)}^{2}\right]}^{1/2}$$

### Statistical analyses

Results were obtained from duplicate of samples, each experimentally quantified in triplicate (each set of three determinations for each duplicate was averaged, thus obtaining two values that were averaged to obtain the values reported as the final quantification value (with its standard deviation), except for color, for which six measurements were done for each duplicate of sample. Statistical data analysis of the results was performed using one‐way Analysis of Variance (ANOVA) and Tukey's HSD Test, at a 5% level of significance.

## Results and Discussion

### Microbial analyses

**Figure 1:**
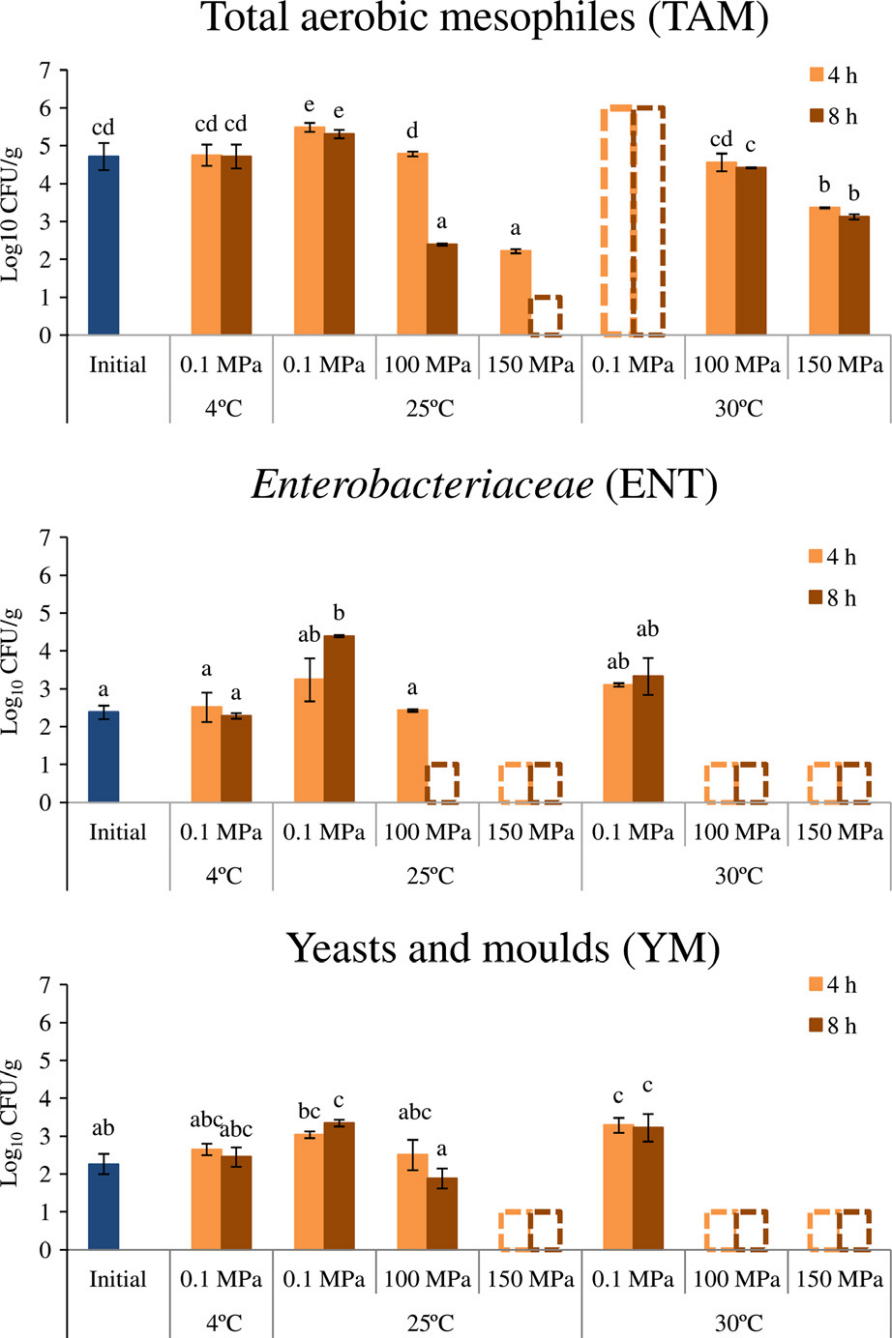
Microbial counts (Log_10_ CFU/g) of TAM, ENT and YM in initial and after hyperbaric storage of soup for various combinations of time, pressure, and temperature: 4 and 8 h, 100 and 150 MPa and 25°C and 30°C; soup was also stored at control conditions for each temperature (25°C and 30°C, at 0.1 MPa) and at refrigeration conditions (4°C, 0.1 MPa). Different letters indicate significant differences (*P* < 0.05) between conditions. Values shown as 6 and 1 log units (bars with discontinuous borders), mean values above (higher than 6 log units), and below (lower than 1 log units) the quantification limits, respectively.

The initial average counts of TAM, ENT, and YM were 4.72 ± 0.36, 2.38 ± 0.17, and 2.26 ± 0.27 Log_10_ CFU/g, respectively (Fig. 1). Refrigeration (4°C, 0.1 MPa) did not cause significant (*P* ≥ 0.05) microbial load changes relatively to the initial counts for both 4 and 8 h of storage for all the studied microorganisms. These results were expected, since it is known that refrigeration slows down microbial growth during storage, maintaining the microbial counts similar to the initial value for the first hours of storage. According to data in the literature, it takes about 6 days until TAM counts exceed the limit of 6.0 Log_10_ CFU/g when soup is stored under refrigeration (Kilinc 2010).

Globally, after storage during 4 and 8 h at atmospheric pressure (0.1 MPa) at 25°C or 30°C, the microbial loads increased significantly (*P* < 0.05) (about 1.0–2.0 Log_10_ CFU/g), comparatively to the initial value, reaching a maximum after storage at 30°C, 0.1 MPa (>6.0 (TAM) and ~3.3 (ENT and YM) Log_10_ CFU/g). These results were also expected since spoilage microorganisms develop relatively fast when products like soup are stored at room temperature, mostly due to the high pH and a_w_ that pose no considerable barriers to microbial growth. When soup was stored under both pressures (100 and 150 MPa), the microbial counts obtained were lower (*P* < 0.05) than those verified for storage at 0.1 MPa, for the same temperature and time storage period (4 and 8 h). Generally, under pressure (HS) after 4 h of storage, the microbial counts obtained revealed a preservation effect similar (*P* ≥ 0.05) to refrigeration, that is, microbial growth inhibition occurred, for all the studied microorganisms, to levels equivalent to refrigeration storage. After 8 h, the microbial counts observed indicated microbial inactivation, since the microbial loads were lower compared to the initial values and the values obtained for refrigeration. These results clearly revealed better performance of HS at 25°C and 30°C compared to refrigeration.

Concerning TAM counts in particular, storage for 4 h at 100 MPa and 25°C caused microbial inhibition (4.79 ± 0.06 Log_10_ CFU/g), resulting in values similar (*P* ≥ 0.05) to refrigeration (4.76 ± 0.28 Log_10_ CFU/g). Under the same storage conditions (100 MPa and 25°C) for 8 h of storage, TAM inactivation was verified (about 2.5 Log_10_ CFU/g), resulting in values lower than refrigeration (*P* < 0.05). Moreover, for storage under 150 MPa at 25°C, it was also observed a clear TAM counts reduction (to 2.22 ± 0.06 Log_10_ CFU/g for 4 h and to below the detection limits for 8 h), this revealing again a microbial inactivation effect, in addition to the microbial growth inhibition effect. For HS at 30°C microbial growth inhibition was observed at 100 MPa (resulting in values similar to refrigeration, *P* > 0.05), while for 150 MPa the additional inactivation effect was verified, with TAM loads reaching values lower than those observed to refrigeration (*P* < 0.05). Interestingly, the effect of HS on TAM was more pronounced at 25°C compared to 30°C. This might be due to the fact that the optimum growth temperature for TAM at atmospheric pressure is 30°C, being probably TAM less susceptible to growth inhibition and inactivation at this temperature.

ENT showed to be more susceptible to HS than TAM, since all the studied HS conditions led to ENT reduction to values below the detection limits, unless for storage at 100 MPa and 25°C for 4 h, where the values (2.43 ± 0.03 Log_10_ CFU/g) remained similar (*P* > 0.05) to refrigeration.

YM counts presented a behavior similar to ENT, since all the studied HS conditions led to microbial reduction to values below the detection limits, unless for storage at 100 MPa and 25°C, for 4 h and 8 h, where the values remained similar (*P* > 0.05) to refrigeration. Interestingly, the effect of HS on YM was more pronounced at 30°C compared to 25°C. This might be due to the fact that optimum growth temperature for YM at atmospheric pressure is 25°C, YM being probably less susceptible to growth inhibition and inactivation at this temperature. A similar situation can be hypothesized for ENT, since these were very susceptible to HS at 25°C and 30°C, while its optimum growth temperature at atmospheric pressure is 37°C, but this hypothesis validation requires HS studies at 37°C.

Globally, the microbial inactivation effect observed (additionally to microbial growth inhibition) with HS was more evident when the soup was stored at high pressure (150 MPa) and for longer storage time studied (8 h). And as already stated, the growth inhibition/inactivation effects observed were also more pronounced at temperatures differing from the optimum growth temperature of each microorganism at atmospheric pressure. Overall, TAM were the microorganisms less susceptible to growth inhibition/inactivation compared to ENT and YM, while the latter showed similar susceptibility.

These results illustrate that hyperbaric storage of soup under 100 and 150 MPa, in the range of room temperatures (25°C and 30°C, and so potentially with no temperature control in a real situation), has potential to replace refrigeration, since the final microbial counts were similar or even lower than those obtained for the soup stored under refrigeration. The microbial inactivation effect observed seems to be dependent on the temperature of storage, and related with the optimum growth temperature at atmospheric pressure for each set of microorganisms under study (however more studies are necessary to confirm this observation).

Results similar to those reported in the present work were observed for storage of raw juices (strawberry, watermelon, and melon juices), where refrigeration kept the microbial loads unchanged (comparatively to initial) during the storage, while storage under pressure allowed not only an inhibition effect for lower pressures (25–100 MPa) but also an inactivation effect for higher pressures (150–220 MPa) for storage at and above room temperature (Segovia‐Bravo et al. 2012; Fidalgo et al. 2014; Queiros et al. 2014).

These results support the possibility to preserve perishable food products under moderate pressure with potentially substantial energy savings, allowing a great reduction in energy costs associated with refrigeration, since HS only needs energy to the compression/decompression phases (being only necessary to reach the required pressure level) and no need to control temperature during storage (contrarily to refrigeration). It should be highlighted that once pressure is generated, there is no need of energy to maintain it. Therefore, in the present work, the results revealed that soup can be preserved at 100 and 150 MPa, with no need for temperature control, with equal, and in some of the studied cases, even better microbial loads compared to refrigeration.

### Physicochemical analysis

#### pH and titratable acidity

Soup's initial pH was 5.65 ± 0.07 which is in agreement with the values reported in the literature that vary between 4 and 6, depending on the type of soup and its composition (Gadekar et al. 2009; Shibeshi and Farid 2011). In Table 1, it is possible to observe a pH stability regardless of pressure, as values are generally not significantly different (*P* ≥ 0.05) from refrigeration. At 30°C, 0.1 MPa, a linear decrease was observed (*R* ^2^ = 0.998; *y* = −0.148*x* + 5.66), during the 8 h of storage, reaching a minimum of 4.46 ± 0.14. These lower values can possibly be the result of the higher microbial loads verified for storage at this condition (Fig. 1).

**Table 1:** pH, titratable acidity, and reducing sugars' values for soup stored for 4 and 8 h at different temperature (°C) and pressure (MPa) conditions. Different letters indicate significant differences (*P* < 0.05) between conditions (a–d) and storage time to the same storage condition (A–B). Values at 4 and 8 h showed at bold are statistically different from the initial value for each parameter and storage condition

Conditions		pH		Titratable acidity (mg malic acid/100 g soup)		Reducing sugars (mg glucose/g soup)	
		4 h	8 h	4 h	8 h	4 h	8 h
Initial		5.65 ± 0.07 bA	5.65 ± 0.07 bcdA	63.65 ± 5.59 abcA	63.65 ± 5.59 bA	6.40 ± 0.32 abA	6.40 ± 0.32 aA
4°C	0.1 MPa	5.57 ± 0.04 bA	5.63 ± 0.17 bcdA	66.20 ± 4.14 abcA	70.04 ± 4.57 bcA	**6.89 ± 0.24** abA	6.72 ± 0.30 aA
25°C	0.1 MPa	5.66 ± 0.20 bA	5.72 ± 0.01 cdA	**50.89 ± 3.15** aA	**53.46 ± 1.43** aA	6.42 ± 0.35 abA	6.10 ± 0.13 aA
	100 MPa	5.54 ± 0.05 b	–a	63.59 ± 3.00 abc	–a	7.56 ± 0.80 b	–a
	150 MPa	5.66 ± 0.01 bA	**5.87 ± 0.02** dB	**55.35 ± 1.85** abA	**47.28 ± 1.12** aB	7.13 ± 0.34 abA	6.85 ± 0.59 aA
30°C	0.1 MPa	**5.10 ± 0.15** aA	**4.46 ± 0.14** aB	**79.44 ± 5.33** cA	**90.50 ± 2.62** dA	**5.97 ± 0.28** aA	**8.01 ± 0.36** bB
	100 MPa	5.61 ± 0.03 bA	**5.44 ± 0.01** bB	65.69 ± 2.11 abcA	**76.06 ± 1.73** cB	6.01 ± 0.50 aA	**9.43 ± 0.19** cB
	150 MPa	5.74 ± 0.12 bA	5.58 ± 0.01 bcA	70.81 ± 8.41 bcA	66.95 ± 1.86 bA	6.13 ± 0.21 abA	6.62 ± 0.29 aA

a Experiments were not carried out in these conditions.

Soup's initial titratable acidity was 63.65 ± 5.59 mg malic acid/100 g soup. The higher values were obtained at 30°C, 0.1 MPa (79.44 ± 5.33 and 90.50 ± 2.62 mg malic acid/100 g soup, after storage for 4 and 8 h, respectively). Titratable acidity in this condition (30°C, 0.1 MPa) tended to linearly increase throughout the 8 h of storage (*R* ^2^ = 0.990; *y* = 3.36*x* + 64.4). These results are in agreement with the lower pH values obtained for storage at 0.1 MPa, 30°C, 8 h. For storage under pressure, the values observed were generally lower, compared to those obtained at the same temperature (25°C or 30°C) at atmospheric pressure and closer to the value obtained with refrigeration.

#### Reducing sugars

In Table 1, it is possible to observe that the reducing sugars' initial value is 6.40 ± 0.32 mg glucose/g soup, and that this value remained stable (*P* ≥ 0.05) after storage for 4 and 8 h at 4°C and 0.1 MPa. The main differences observed between the different storage conditions and the initial values for reducing sugars content were: higher values for storage at 25°C, 100 MPa, 4 h and for storage at 30°C, 0.1 and 100 MPa, 8 h. These results are probably due to the presence of a higher microbial load in these samples that might lead to starch hydrolysis (from the potatoes used to prepare the soup), thus increasing the amount of reducing sugars.

#### Color

The soup at the beginning of the study presented a bright orange color (L* = 52.44 ± 1.07), tending to red (a* = 4.35 ± 0.16) and yellow (b* = 33.03 ± 2.12) (see Table 2). The L* parameter (lightness) remained stable after storage at 4°C, 0.1 MPa. For storage at 30°C, 0.1 MPa, L* increased linearly (*R* ^2^ = 0.997; *y* = 0.368*x* + 52.5) with storage time, being verified a maximum of 55.39 ± 0.56 after 8 h. On the other hand, when soup was stored under pressure, the L* value did not significantly change (*P* ≥ 0.05) comparatively to refrigeration, except for storage at 25°C, for 4 h, at 150 MPa, where a minimum of 46.17 ± 0.41 was reached. In general, the higher variations for L* were observed for the samples stored at 0.1 MPa. For the redness parameter (a*), a slight increase was observed (*P* ≥ 0.05) after refrigerated storage (4°C, 0.1 MPa), comparatively to the initial value. Storage at 0.1 MPa, for 4 h, either at 25°C or 30°C, did not cause significant variations (*P* ≥ 0.05), while after 8 h of storage this value increased relatively to the initial value. For HS, the main differences observed were a higher value (*P* < 0.05) of 6.09 ± 0.07 for 25°C, 100 MPa and 4 h, and a lower value (*P* < 0.05) for 30°C, 100 MPa, 8 h (4.24 ± 0.24) compared to refrigeration, but not statistically different from the initial value. The b* parameter (yellowness) remained generally stable (*P* > 0.05) for all the conditions.

**Table 2:** Color parameters for soup stored for 4 and 8 h at different temperature (°C) and pressure (MPa) conditions. Different letters indicate significant differences (*P* < 0.05) between conditions (a–c) and storage time to the same storage condition (A–B). Values at 4 and 8 h showed at bold are statistically different from the initial value for each parameter and storage condition

Conditions		Color							
		L*		a*		b*		ΔE*	
		4 h	8 h	4 h	8 h	4 h	8 h	4 h	8 h
Initial		52.44 ± 1.07 cA	52.44 ± 1.07 aA	4.35 ± 0.16 abA	4.35 ± 0.16 aA	33.03 ± 2.12 abA	33.03 ± 2.12 abA	–	–
4 °C	0.1 MPa	53.60 ± 1.34 cA	53.43 ± 0.88 abA	**5.36 ± 0.34** bcA	4.85 ± 0.22 bcA	34.94 ± 0.91 abA	34.13 ± 1.15 abA	3.21 ± 1.12	1.77 ± 0.67
25°C	0.1 MPa	**49.46 ± 0.28** bA	52.68 ± 1.19 aB	4.37 ± 0.11 abA	**5.52 ± 0.51** cB	31.68 ± 0.19 aA	**35.43 ± 1.85** bB	5.65 ± 0.18	7.53 ± 2.20
	100 MPa	53.70 ± 0.24 c	–a	6.09 ± 0.07 c	–a	37.91 ± 0.24 b	–a	2.65 ± 0.01	–a
	150 MPa	**46.17 ± 0.41** aA	52.25 ± 0.09 aB	**4.91 ± 0.13** abcA	**4.72 ± 0.18**abA	33.31 ± 1.34 abA	33.94 ± 0.43 abA	6.27 ± 0.41	5.71 ± 1.14
30°C	0.1 MPa	54.05 ± 1.68 cA	**55.39 ± 0.56** cA	4.40 ± 0.65 bcA	5.24 ± 0.42 bcA	32.83 ± 2.53 abA	35.52 ± 1.26 bA	1.74 ± 0.50	3.99 ± 1.10
	100 MPa	52.49 ± 1.42 cA	52.35 ± 0.38 aA	4.04 ± 0.78 abA	4.24 ± 0.24 aA	32.41 ± 1.71 aA	32.27 ± 0.57 aA	1.53 ± 0.49	1.27 ± 0.80
	150 MPa	**51.56 ± 0.28** bcA	**54.23 ± 0.01** bcB	**3.60 ± 0.15** aA	4.80 ± 0.21 abB	30.14 ± 0.53 aA	34.04 ± 0.23 abB	2.89 ± 0.72	2.19 ± 0.10

a Experiments were not carried out in these conditions.

A noticeable difference between food products global color state is considered perceptible by the consumer when they differ by a total color difference, ΔE*, higher than 2.0–3.5 (Krapfenbauer et al. 2006). The ΔE* values of soup stored at 0.1 MPa, 25°C, and 30°C were higher than 3.5 (Table 2), indicating that storage in these conditions caused a visible color difference. It is noteworthy that after storage under 100 MPa, for both 4 and 8 h, the ΔE* values remained below the limit established to be perceived by the consumer as a color difference.

## Conclusion

Hyperbaric storage (HS) of a RTE soup in the range of room temperatures (25°C and 30°C) proved to be a preservation method capable not only to inhibit microbial growth at levels similar to refrigeration but even to inactivate microorganisms, leading to lower microbial loads at the end of the storage compared to refrigerated storage. Microbial growth inhibition prevailed at 100 MPa and 4 h of storage, while microbial inactivation was more evident at 150 MPa (4 and 8 h of storage) when compared to the initial value of the fresh soup. For so, the storage condition that allowed better preservation of soup characteristics, while maintained microbial safety was HS 150 MPa, at 25°C, for 8 h. HS maintained, generally the pH, titratable acidity, reducing sugars and color at values closer to refrigeration, compared to storage at 0.1 MPa.

In conclusion, hyperbaric storage is a methodology with great potential as a potential alternative to refrigeration as a food preservation technology, basically energetically costless. Other studies are of interest to advance the scientific knowledge in this area, like the study of longer storage times, different pressure/temperature combinations, other microbial populations, and other food quality‐related parameters. Also economical studies, taking into account energy requirements and equipment costs, should be further carried out to evaluate the practical feasibility of the use of this novel food preservation methodology.

## Conflict of Interest

None declared.
